# How to utilize Ca^2+ ^signals to rejuvenate the repairative phenotype of senescent endothelial progenitor cells in elderly patients affected by cardiovascular diseases: a useful therapeutic support of surgical approach?

**DOI:** 10.1186/1471-2482-13-S2-S46

**Published:** 2013-10-08

**Authors:** Francesco Moccia, Silvia Dragoni, Mariapia Cinelli, Stefania Montagnani, Bruno Amato, Vittorio Rosti, Germano Guerra, Franco Tanzi

**Affiliations:** 1 Department of Physiology, University of Pavia, via Forlanini 6, 27100 Pavia, Italy; 2 Department of Public Health, University of Naples "Federico II", via Pansini 5, 80131, Naples, Italy; 3 Department of Clinical Medicine and Surgery, University of Naples "Federico II", via Pansini 5, 80131, Naples, Italy; 4 Unit of Clinical Epidemiology, Fondazione IRCCS Policlinico San Matteo, 27100 Pavia, Italy; 5 Department of Medicine and Health Sciences, University of Molise, Via F. De Santis, 86100 Campobasso, Italy

## Abstract

Endothelial dysfunction or loss is the early event that leads to a host of severe cardiovascular diseases, such as atherosclerosis, hypertension, brain stroke, myocardial infarction, and peripheral artery disease. Ageing is regarded among the most detrimental risk factor for vascular endothelium and predisposes the subject to atheroscleorosis and inflammatory states even in absence of traditional comorbid conditions. Standard treatment to restore blood perfusion through stenotic arteries are surgical or endovascular revascularization. Unfortunately, ageing patients are not the most amenable candidates for such interventions, due to high operative risk or unfavourable vascular involvement. It has recently been suggested that the transplantation of autologous bone marrow-derived endothelial progenitor cells (EPCs) might constitute an alternative and viable therapeutic option for these individuals. Albeit pre-clinical studies demonstrated the feasibility of EPC-based therapy to recapitulate the diseased vasculature of young and healthy animals, clinical studies provided less impressive results in old ischemic human patients. One hurdle associated to this kind of approach is the senescence of autologous EPCs, which are less abundant in peripheral blood and display a reduced pro-angiogenic activity. Conversely, umbilical cord blood (UCB)-derived EPCs are more suitable for cellular therapeutics due to their higher frequency and sensitivity to growth factors, such as vascular endothelial growth factor (VEGF). An increase in intracellular Ca^2+ ^concentration is central to EPC activation by VEGF. We have recently demonstrated that the Ca^2+ ^signalling machinery driving the oscillatory Ca^2+ ^response to this important growth factor is different in UCB-derived EPCs as compared to their peripheral counterparts. In particular, we focussed on the so-called endothelial colony forming cells (ECFCs), which are the only EPC population belonging to the endothelial lineage and able to form capillary-like structures *in vitro *and stably integrate with host vasculature *in vivo*. The present review provides a brief description of how exploiting the Ca^2+ ^toolkit of juvenile EPCs to restore the repairative phenotype of senescent EPCs to enhance their regenerative outcome in therapeutic settings.

## Introduction

Senescence and aging involving several mechanisms like oxidative stress and elevated ROS (Reactive oxygen species). They has been implicated in cancer, diabetes, neurodegenerative, cardiovascular and other diseases [1,2]. Several stressors, including high-caloric diets, physical activity, chemicals, drugs and pollutants, induce oxidants overproduction [3]. The endothelium forms a multifunctional signal transducing surface that lines the luminal surface of both blood vessels and cardiac chambers, thereby maintaining cardiovascular homeostasis [4]. Such strategic location places endothelial cells (ECs) in the most suitable condition to properly govern blood pressure, coagulation and fibrinolysis, vascular inflammatory reactions, permeability of the vessel wall, and angiogenesis [5,6]. The endothelial monolayer modulates such different functions due to its ability to synthesize and release a myriad of agents that regulate vasomotor function, trigger inflammatory processes, and affect haemostasis [5,7]. Nitric oxide (NO), prostacyclin (PGI_2_), carbon monoxide (CO), hydrogen sulphide (H_2_S), epoxyeicosatrienoic acids (EETs), and adenosine represent the main vasodilatory factors produced by vascular ECs [7,8], while vasoconstrictors include endothelin-1 (ET-1), angiotensin II (Ang II), thromboxane A2 (TXA_2_), prostaglandin H2 (PGH_2_), and reactive oxygen species (ROS) [6,8]. Furthermore, ECs directly communicate with the underlying smooth muscle cells (SMCs) through myoendothelial gap junctions which spread endothelial-dependent hyperpolarization (the so-called endothelium-dependent hyperpolarizing factor, EDHF) and reduce the vascular tone [9]. In addition, cardiac microvascular ECs adjust myocardial contractility according to incoming inputs by releasing paracrine mediators, such as NO, ET-1 and prostanoids [10]. As a consequence, endothelial damage attenuates the vaso-relaxing, anti-thrombotic, and anti-inflammatory properties of the endothelial sheet and causes a shift to conditions prone to vasoconstriction, coagulation, and inflammation [6,8]. This chain of events depends on reduction in NO bioavailability and the alterations in the production of most, if not all, the humoral and electrical factors described above [6,11]. In addition, vascular ECs prevent SMC proliferation by liberating several growth inhibitors, such as NO and prostacyclins [12,13]. Endothelial dysfunction may thus promote vessel thickening by stimulating SMCs to switch from a non-contractile phenotype and migrate from the tunica media towards the inner lumen [11,12]. This process induces the inward remodeling of vascular architecture, with variable degrees of arteriolar narrowing in the chronic phase, the so-called restenosis [14]. Overall, endothelial dysfunction leads to the impairment of blood perfusion to vital and peripheral organs and compromises cardiovascular homeostasis [6,11-13]. This leads to the development and progression of potentially lethal cardiovascular diseases, such as hypertension, atherosclerosis, atherothrombosis, myocardial infarction, brain stroke and vascular forms of renal failure [6,11].

Tissue perfusion through stenotic supplying arteries in patients suffering symptomatic occlusive atherosclerosis or surviving to the acute phase of a heart attack may be restored by a number of surgical approaches. These include angioplasty, deployment of intracoronary stents, percutaneous coronary intervention (PCI), and coronary artery bypass surgery. These procedures, however, may further affect the integrity of the endothelial monolayer and, in the long term, may cause thrombi formation and neointimal hyperplasia. The subsequent shrinkage of the arterial wall severely limits the beneficial outcome of reconstructive surgery and leads to in-stent restenosis-related heart disease [15-17]. In addition, ischemia resulting from microvascular or endothelial dysfunction results in obstructive peripheral artery disease (PAD), a major cause of morbidity and mortality in the western world, by accounting for up to 30% of deaths worldwide [16]. In its most advanced form, critical limb ischemia (CLI), the insufficient blood flow to lower extremities is associated to atypical leg pain and intermittent claudication, and may progress to tissue lost, gangrene and amputation when either surgical or endovascular revascularisation fail to restore local perfusion [16,17]. The wide array of unmet medical needs in the treatment of cardiovascular diseases boosted the search for alternative strategies to stimulate therapeutic angiogenesis by recapitulating the vascular network within the ischemic organ [4-6]. The adult bone marrow (BM) is a rich reservoir of progenitor and stem cells, which possess the capability for self-renewal and differentiation into organ-specific cell types [7]. As shown in several animal models, transplantation of these cells may reconstitute organ systems, a therapeutic approach termed cell-based therapy (CBT). The discovery that non-resident cells contribute to vascular repair paved the way for unexpected therapeutic options, since these cells can be harvested, expanded and re-inoculated to boost vascular regeneration [16-19]. It has now been established that endothelial progenitor cells (EPCs) are among the most suitable bone marrow-derived cell populations to regenerate ischemic organs and hold great promise to meet the untreatable clinical demands of cardiovascular disorders [20-24]. Unfortunately, both the frequency and the pro-angiogenic activity of circulating EPCs are dramatically impaired in the elderly, thereby limiting the use of autologous CBT in ageing population [25,26]. Recent work conducted by our group has disclosed the master role served by intracellular Ca^2+ ^signalling in controlling EPC proliferation, homing and assembly into capillary-like structures. The present review will briefly address the possibility to exploit specific components of the Ca^2+ ^toolkit to improve the regenerative outcome of EPC-based therapy in ageing patients.

## Ageing is relevant risk factor for vascular endothelium: causes and detrimental impact on the outcome of therapeutic treatment of ischemic diseases

Ageing is regarded among the most detrimental threatens for vascular endothelium and, therefore, human health. The impairment of EC function is a progressive phenomenon that starts in the middle age, has been termed endothelial senescence and may occur in absence of any other cardiovascular risk factor [25-27]. Age-related endothelial dysfunction is associated to deterioration in the balance between vasodilator and vasoconstriction mediators liberated by vascular ECs. In females postmenopausal hypoestrogenism may cause an increase in arterial vascular tone through a reduction of vasodilator peptides and an increase in vasoconstrictor peptides in the arterial-wall termination of the autonomous system in some anatomical districts leading several symptoms. These changes in neuropeptide content in the arterial walls might represent a new mechanism underlying the negative effects of menopause on the cardiovascular system [28-30]. Vascular ECs imbalance is featured by a progressive decrease of NO bioavailability and an enhanced production of cyclooxygenase-derived vasoconstrictor factors, such as TXA_2 _and PGH_2 _[6,31]. Furthermore, a decline in EDHF contribution to endothelial dilatation of human arteries comes about in different vascular regions [26], albeit studies conducted on murine models do not conclusively support this notion [25]. The decrease in endothelial NO synthesis with ageing is paralleled by an elevation in O_2 _production and in the subsequent generation of ROS, pivotal among which are the superoxide radical (O_2•_^-^) and the peroxynitrite anion (ONOO^-^) [25,26]. The severe oxidative stress imposed on the endothelial monolayer in elderly contributes to rapidly inactivate NO, which may be scavenged by O_2•_^-^, and is accompanied by a low-grade chronic inflammatory state, thereby originating the so-called "inflammaging", which involves up-regulation of cellular adhesion molecules, an increase in endothelial-leukocyte interactions and permeability, as well as alterations in the secretion of autocrine/paracrine cytokines, which are pivotal to inflammatory responses [25,32]. Among these, an increase in the circulating levels of TNF-α, IL-1β, and members of the super-family of IL-6, as well as of mitochondrial-derived ROS, is the *primum movens *for the nuclear factor-kappa B (NF-*k*B)-mediated transcription of endothelial pro-inflammatory genes that lead to vascular dysfunction [33]. It is, therefore, not surprising that age-related oxidative stress and inflammation may act as powerful pro-atherogenic factors even in the absence of any other cardiovascular risk factor, such as smoking, hypertension, obesity, diabetes, and so on [25,26,33]. Consistent with the pro-atherosclerotic remodelling of the vascular structure, senescent ECs undergo a dramatic alteration in their morphological phenotype, by adopting an enlarged and flattened morphology and displaying organelle hyperplasia [34]. Furthermore, they express senescence-associated enzymes, such as the acidic β-galactosidase (SA-β-gal). Last, but not least, ageing ECs loose their capability to proliferate and to substitute adjacent/neighbouring damaged cells, thereby aggravating the impact of endothelial detachment from the vessel wall, that may be provoked by turbulent blood flow, higher incidence of apoptosis or iatrogen interventions [31]. The exit from the cell cycle and the permanent growth arrest are due to an impaired telomerase activity or telomere shortening as a result of the "inflammaging" state suffered by the vascular wall [11,25,31]. The limited capacity for endothelium to regenerate the denuded vascular wall and to undergo angiogenesis renders elderly patients less prone to receive aggressive coronary revascularisation and augments the risk of adverse outcomes with PCI, i.e. late in-stent restenosis [15]. Moreover, elderly patients with symptomatic ischemic heart disease may not be amenable for surgical revascularization due to accompanying comorbidities or small vessel disease [16,17]. Likewise, the strengths and limitations of surgical revascularization in elderly patients affected by obstructive vascular diseases are well known. They are, therefore, left with few, if any, effective therapeutic methods to alleviate symptom, restore distal perfusion and preserve tissue viability [15-17]. Therapeutic angiogenesis represents an alternative strategy to generate new blood vessel growth, thereby promoting neovascularization and tissue repair, in patients who are not amenable for vascular reconstructive surgery [16,17,35]. Currently, there are three major indications for which angiogenic therapies are in clinical use: 1) chronic wounds (e.g. diabetic lower extremity ulcers, venous leg ulcerations, pressure ulcers, arterial ulcers); 2) PAD; and 3) ischemic heart disease. In such conditions, the therapeutic goal is to stimulate angiogenesis to improve perfusion, deliver survival factors to sites of tissue repair, mobilize regenerative stem cell populations, and ultimately, restore form and function to the tissue [16-18,35,36]. Cellular-based strategies are now referred to as the most promising approach to treat disorders of inadequate blood perfusion by injecting the most suitable cell population to recapitulate the damaged vascular network [16-18,22,36]. We refer the reader to a number of exhaustive and recent review describing the most popular cell populations and methods of delivery utilized in both pre-clinical studies and clinical trials [16-18,24,27,36-39]. In the following paragraphs, we will focus on circulating EPCs and will speculate on the possibility to utilize their Ca^2+ ^machinery to overcome the hurdles related to EPC senescence and improve the outcome of CBT.

## Endothelial progenitor cells as an alternative tool to restore blood perfusion in ischemic tissues: umbilical cord blood as a better cell source as compared to aged peripheral blood

Endothelial progenitor cells (EPCs) are mobilized from bone marrow to replace the ECs sloughed off from the vascular wall as a result of either apoptosis or a traumatic vascular injury [20-24,40]. Not only EPCs physically integrate within the lesioned monolayer. They do stimulate local angiogenesis by the paracrine release of growth factors and chemokines, such as vascular endothelial growth factor (VEGF) and stromal derived factor-α (SDF-α), that induce ECs from adjacent capillaries to sprout towards the ischemic area [20-24,40]. Under normal conditions, EPCs are embedded in a microenvironment (niche) of BM, so that the number of circulating EPCs is relatively small in absence of any cardiovascular trauma. Vascular insult or disease causes the up-regulation of the hypoxia inducible factor α (HIFα), a transcription factor driving the local expression of the cytokines - VEGF, SDF-α, and erythropoietin (EPO) - that stimulate EPC release from the stem cell niche into peripheral blood (PB). EPCs in turn follow the cytokine gradient to the site of arterial injury, where they adhere to the denuded extracellular matrix and replace lost cells by acquiring a mature endothelial phenotype [22,23,41]. Unfortunately, the term EPC does not refer to a unique cell population, with an identified panel of surface antigens or makers or a well characterized transcriptomic profile; currently, EPC is a definition that encompasses a broad range of cell subsets, which may all stimulate angiogenesis *in vivo*, but are not necessarily truly committed endothelial progenitors. We refer the reader to a number of recent reviews addressing the phenotypic and functional characterization of the three main populations of putative circulating EPCs, i.e. colony forming units-ECs (CFU-ECs), circulating angiogenic cells (CACs), and endothelial colony forming cells (ECFCs) [20,21,23,40,41]. CFU-ECs and CACs belong to the hematopoietic lineage and are able to induce therapeutic angiogenesis by paracrine assistance of local ECs via the production of VEGF and SDF1-α. Conversely, ECFCs express all the typical surface antigens of the endothelial lineage (CD105, CD31, CD144, von Willebrand factor, VEGFR-2) but not the CD45 and CD14 antigens, which feature the hematopoietic phenotype. In addition, ECFCs exhibit a robust clonogenic proliferative capacity and assembly into capillary-like networks both *in vitro *and *in vivo*. In particular, ECFCs stably integrate within the vasculature of the host animal, thereby permitting blood flow, when suspended in a collagen scaffold and transplanted into immunodeficient mice [20-23,40-43]. Therefore, ECFCs are regarded as the most suitable EPC subtype to replace lost ECs and to promote vascular healing in clinical settings. It should, however, be pointed out that ECFCs are not only mobilized from BM; a complete hierarchy of highly proliferative ECFCs has indeed been described in the vessel wall of human arteries [20], albeit their contribution to endothelial repair *in vivo *has not been experimentally verified.

Once understood that pre-clinical and clinical studies conducted to assess the regenerative potential of circulating EPCs are largely heterogeneous as respect to their identification, characterisation and functional role, an evident link has been established between the frequency of EPCs in peripheral blood and the propensity for cardiovascular disease. A decrease in the level of circulating (CD34^+ ^VEGFR-2^+^) EPCs is a reliable and independent predictor of an increased risk for atherosclerosis, and has been associated to the increase in intima-media thickness that features the atherosclerotic lesions in distinct vascular districts [18,23,38]. Similarly, lower CD34^+^/CD133^+^/VEGFR-2^+ ^EPC counts have been reported in patients with multivessel coronary artery disease [23,37,38]. Likewise, a rapid and significant increase in EPC numbers, detected by utilising 5 different approaches (CD34^+^, D34^+^/CD117^+^, CD34^+^/CXCR4^+^, CD34^+^/CD38^+ ^and CD34^+^/CD45^+^), has been reported after the onset of ischemia in acute myocardial infarction [22,38]. Notably, the higher frequency of progenitor cells was positively correlated with an improvement of ventricular function and negatively correlated with myocardial necrosis markers in at least 2 of these studies [22]. In agreement with hypoxia acting as a primary stimulator of EPC mobilization, a significant rise in EPC (CD34^+^/CD133^+^/VEGFR-2^+^) numbers occurs in patients with unstable angina and in mild heart failure (NYHA class I-II), whereas their release in circulation is dampened in subjects with advanced heart failure (NYHA III-IV). At the same way, CD34^+^/CD133^+^/VEGFR-2^+ ^EPCs increase during the moderate phase of PAD, while they significantly decrease below the control levels in the advanced phase [44]. These observations have been accompanied by a myriad of pre-clinical studies and clinical trials which were devoted to assess the feasibility of utilising EPCs in regenerative therapy. In more detail, it was demonstrated that: 1) *ex vivo *expanded human EPCs increased capillary density and reduced the rate of limb loss when implanted into murine models of hind limb ischemia; 2) exogenously administered EPCs increased the extent of neovascularisation and improved the left ventricular ejection fraction (LVEF) when injected intravenously into rats with myocardial ischemia; 3) infusion of autologous EPCs restored endothelial functions and improved vascularisation in patients suffering from PAD and limb ischemia, respectively; 4) transplantation of PB-derived EPCs in patients who underwent AMI or suffered from chronic myocardial ischemia led to an improvement in LVEF, a reduction in infarct size, and an increase in capillary density; 5) EPCs-capturing stents, which are based on CD34 antibody coated device, accelerate the re-endothelialization of the damaged arterial segment and reduce the risk of in-stent restenosis after PCI in both animal models and in human subjects [18,24,36-39,45,46]. On the basis of these evidences, EPCs has been put forward as the most promising novel therapeutics of peripheral and myocardial ischemia. Several hurdles are, however, associated to this kind of approach. The enthusiasm raised by pre-clinical studies has been somehow dampened by clinical practice, according to which the extent of cardiac repair upon EPC inoculation is inadequate as compared to animal models. For example, intra-artery delivery of CD133^+ ^EPCs after an acute coronary syndrome led to a 5% elevation in LVEF and reduced volume expansion beyond that provided by state-of-the-art surgical and/or pharmacological therapy as assessed by subsequent meta-analyses [18,19,22,47]. The survival benefit of EPC infusion on patients surviving to AMI is, therefore, much less impressive than expected from regenerative therapy, albeit clinically helpful. Moreover, injection of total BM mononuclear cells into the heart of infarcted rats has been shown to induce severe intramyocardial calcifications in a pre-clinical study [39]. Moreover, EPCs have been shown to transdifferentiate into smooth muscle cells *in vivo*, thereby contributing to enhance intimal hyperplasia upon balloon injury [39,48]. These evidences indicate that EPCs may acquire a phenotype other than the endothelial lineage, a feature which may potentially hamper their regenerative efficacy and harm patient health. The current limitations in autologous EPCs-based therapy have so far been ascribed to a number of causes, which include: 1) the amount of blood volume (12 L) required to isolate a therapeutically sufficient amount of EPCs (0.5~2.0 ⋯ 10^4 ^human EPCs/g body weight) [45]; 2) the low percentage (10%) of EPCs retention and survival within the injury site [19,22]; 3) the hostile microenvironment in the target organ after AMI, characterized by inflammation, fibrosis, and inadequate angiogenesis [27]; 4) the variability in the EPC subtype that has been transplanted into ischemic patients; 5) our poor comprehension of the molecular mechanisms driving EPC proliferation, homing and differentiation into mature ECs *in vivo*, as most of the studies in the filed have been carried out to evaluate on their potential application in medicine, rather than to shed light on their biology. Finally, all clinical trials enrolled patients with AMI who had undergone primary angioplasty and stent implantation to re-expand the infarct-related artery, and cells were delivered intra-coronary by using the stop-flow balloon catheter strategy. In this regard, the clinical studies differed significantly from the animal studies, where the artery was not reperfused and cells were directly injected into the myocardium. In addition, preclinical animal models, apart from the ischemic injury caused by ligation of the coronary artery, are usually young and healthy. Conversely, human subjects with chronic ischemic diseases (end-stage heart failure and PAD) suffer from a number of co-morbidities that may attenuate the innate reparative properties of stem cells. Indeed, ageing and male individuals have lower CD34^+^/VEGFR-2^+ ^EPCs than their younger counterparts [25,26]; moreover, the pro-angiogenic activity of senescent EPCs is severely impaired, the decline in their clonogenic potential occurring at an earlier age as respect to their migratory activity [49]. It is, therefore, not surprising that 1) EPCs harvested from older rats failed to promote neovascularisation in a corneal micropocket assay implanted into younger mice and 2) EPCs isolated from older patients with clinical ischemia were less effective in rescuing mice hind limb ischemia as compared to those from younger ischemic patients [27,50]. It appears that the most promising strategy to harness autologous EPCs in CBT is to rejuvenate their regenerative potential by genetically intervening on the signal transduction pathways driving cell proliferation, migration, and differentiation [51,52]. Enhancing their capability of perceiving and translating pro-angiogenic inputs, such as those provided by VEGF and SDF1-α, into a therapeutically relevant biological response will open a new avenue in the treatment of cardiovascular diseases in elderly. This requires a careful comparison of the molecular mechanisms responsible for DNA synthesis and motility between PB-derived EPCs and EPCs harvested from umbilical cord blood (UCB), which display a higher proliferation capacity and express higher levels of telomerase. In addition, the choice of the most effective reparative phenotype, while ensuring optimal cell delivery, dosing, and timing of intervention, is essential to ensure the successful outcome of such novel therapeutic approach. ECFC stand out among the most suitable candidates for cellular-based therapeutics due to their ability to stimulate local angiogenesis through paracrine signalling and physically engraft within neovessels. Central to the goal of the present review, UCB-derived ECFCs (UCB-ECFCs) are much more amenable to CBT than their peripheral counterparts (PB-ECFCs). First, ECFCs are much more abundant in UCB than in PB (2.5 cells/ml *vs*. 0.05-0.2 cells/ml), so that adequate numbers of cells may be obtained to treat ischemic diseases in human patients [20,53]. Second, telomerase activity is much more prominent in UCB-ECFCs, thereby delaying their entrance into the senescence state and enhancing their regenerative potential [20,53]. Third, the proliferative response to VEGF is dramatically higher in UCB-ECFCs as compared to PB-derived cells, with up to 1000 population doublings observed *vs*. 20-30 [53]. Consistent with these observations, EPCs engineered to overexpress VEGF displayed higher proliferative and adhesion capabilities *in vitro *than non-transduced cells [51,52]. In addition, when these cells were injected into a murine model of hind limb ischemia, both the rate of neovascularisation and the recovery of blood flow were significantly improved as compared with controls [51,52]. Similarly, transplantation of VEGF gene-transduced EPCs accelerated organization and recanalization of venous thrombi by increasing capillary density at this location [51,52]. A remarkable feature of combining gene- and cell-based therapies concerns the number of cells employed to repair the damaged tissue. Indeed, the dose of VEGF-overexpressing EPCs required to achieve limb savage was 30 times lower than that utilised in previous experiments conducted with naïve cells [51,52]. These findings support the notion that the genetic manipulation of the signaling machinery that governs EPC bioactivity alleviates potential age-related EPC dysfunction and meet therapeutic needs.

## The Ca^2+ ^signaling toolkit as a novel molecular target to enhance the regenerative potential of senescent endothelial colony forming cells

It has long been known that an increase in intracellular Ca^2+ ^concentration ([Ca^2+^]_i_) is a critical node in the signalling network that governs the endothelial response to both chemical and mechanical stimuli [54,55]. In particular, calcium ions play a master role in the complex and multistepped process of angiogenesis by regulating endothelial proliferation, migration, adhesion to the substrate, contractility and organization into capillary-like structures [7,22,23,54]. The tight relationship between Ca^2+ ^dynamics and endothelial signalling is not surprising when considering that Ca^2+ ^functions as a ubiquitous intracellular second messenger involved in a myriad of processes as diverse as fertilization, gene transcription, bioenergetics, secretion, controlled cell death, and so on [56]. Endothelial Ca^2+ ^signals consist in spatially and temporally defined changes in [Ca^2+^]_i _that represent stimulus-specific Ca^2+ ^signatures. These Ca^2+ ^signatures are detected, decoded and relayed to the target enzyme by a complex toolkit of Ca^2+^-binding proteins, pivotal among which are calmodulin (CaM), Ca^2+^/CaM-dependent kinases (CaMKI, CaMKII and CaMKIV) and calcineurin, that act as Ca^2+ ^sensors [22,23,54]. Compartmentalization is the keyword to understand the versatility of Ca^2+ ^signalling. The Ca^2+^-dependent mediators are indeed physically placed in close proximity of the cytosolic mouth of the Ca^2+ ^channel providing the source of the triggering signal [56-58]. This arrangement of the Ca^2+ ^machinery enables one single ion to regulate a multitude of even opposite cellular processes depending of the Ca^2+^-permeable conductance engaged by extracellular stimulation. Vascular ECs, as well as other non-excitable cell types, deliver Ca^2+ ^signals in the form of brief transients which rapidly decay to the baseline [54,59,60]. A prolonged elevation in [Ca^2+^]_i _may, indeed, be detrimental for the cell since a massive Ca^2+ ^load may either induce the pro-apoptotic cascade or cause necrosis by stimulating both Ca^2+^-dependent proteases, such as calpain, and endonucleases [61]. Therefore, long-term cellular responses, such as activation of the transcriptional programme driving cell proliferation and differentiation, are regulated by a train of cytoplasmic Ca^2+ ^oscillations that persist as long as the cells are presented with the growth factor [48,54,58]. A heterogeneous increase in [Ca^2+^]_i _is the hallmark that features endothelial monolayers exposed to either VEGF or EGF, whose pro-angiogenic effect is suppressed when the intracellular Ca^2+ ^signal is abolished [62,63]. This firmly established notion prompted our group to investigate for the first time whether and how VEGF utilizes the Ca^2+ ^machinery to promote EPC proliferation and tubulogenesis *in vitro*. In particular, we focussed our efforts on ECFCs isolated from both peripheral and umbilical cord blood with the aim to search for differences in the components of the Ca^2+ ^toolkit selected by VEGF in the two cell types. At the beginning, we found that PB-ECFCs exposed to VEGF generate asynchronous oscillations in [Ca^2+^]_i _which arise even in the absence of extracellular Ca^2+ ^influx [64]. Nevertheless, Ca^2+ ^entry is essential to maintain the repetitive Ca^2+ ^spike over time. The signal transduction pathway that orchestrates the oscillatory response is initiated by the plasma membrane-bound enzyme, phospholipase Cγ (PLCγ), which is enlisted by VEGFR-2 to cleave phosphatidylinositol-4,5-bisphosphate (PIP_2_), a minor phospholipid precursor, into inositol-1,4,5-trisphosphate (InsP_3_) and diacylglycerol (DAG). InsP_3 _diffuses within the cytoplasm and binds to Ca^2+^-permeable InsP_3 _receptors (InsP_3_Rs) located in the endoplasmic reticulum (ER) membranes, the largest intracellular Ca^2+ ^reservoir. The binding of InsP_3 _induces a conformational change in InsP_3_R structure, thereby causing the rhythmic Ca^2+ ^release that shapes the first Ca^2+ ^transients. PB-ECFCs possess all the 3 known InsP_3_R isoforms, i.e. InsP_3_R-1, InsP_3_R-2, and InsP_3_R-3, the pattern of expression being InsP_3_R-3>InsP_3_R-2>InsP_3_R-1 [64]. The biphasic dependence of InsP_3_R gating by ambient Ca^2+ ^(<500 nM activate it, >1 μM inhibit it) is the most likely responsible for the cyclic opening and closing of the channel pore in spite of the constant levels of cytosolic InsP_3 _[65,66]. Alternatively, PLCγ might undergo a PKC-induced inhibition, which would provide the feedback mechanism underpinning the periodic mobilization of intralumenal Ca^2+ ^by InsP_3 _[66]. The following drop in ER Ca^2+ ^signals the activation of a Ca^2+^-permeable conductance on the plasma membrane, which has universally been termed as store-operated Ca^2+ ^entry (SOCE) or capacitative Ca^2+ ^entry (CCE) [64,67]. Not only SOCE is the most important pathway for Ca^2+ ^influx in vascular endothelium, which normally lacks voltage-gated Ca^2+ ^channels to replenish its intracellular Ca^2+ ^pools [54]. It is the only source for Ca^2+ ^inflow downstream PLC activation in PB-ECFCs, which are devoid of the DAG-sensitive Canonical Transient Receptor Potential (TRPC) channels 3 (TRPC3), TRPC6 and TRPC7 [67,68]. SOCE is mediated by the physical interplay between Stromal Interaction Molecule-1 (Stim1), the ER Ca^2+ ^sensor, which oligomerizes and redistributes into sub-membranal clusters, referred to as *puncta*, in response to ER emptying. Herein, Stim1 activates a Ca^2+^-entry route which is contributed by both Orai1 and TRPC1, albeit it is unclear whether they all assemble into a heteromeric ternary complex or whether TRPC1 and Orai1 form two distinct store-operated channels [22,64,67-69]. The interplay between InsP_3_-dependent Ca^2+ ^discharges and SOCE shapes the oscillatory Ca^2+ ^response to VEGF we observed in circulating ECFCS. NF-κB provides the mechanistic link between the train of Ca^2+ ^transients elicited by VEGF and its stimulatory effect on PB-ECFC proliferation and tubulogenesis in Matrigel plugs [64]. In absence of extracellular stimulations, NF-κB is retained in the cytosol by the complex with the inhibitory protein, I_kB_, which masks its nuclear localization signals (NLS) [22]. Oscillations in [Ca^2+^]_i _promote a phosphorylation cascade which is mediated by Ca^2+^/CaM-dependent protein kinases and leads I_kB _to site-specific ubiquitination and subsequent degradation. Consequently, NF-κB is released from inhibition and translocates into the nucleus, where it activates the transcriptional programme responsible for cell survival and proliferation [22]. Earlier work has suggested that periodic Ca^2+ ^signals encode information in a 'digital' manner, whereby the increasing strength of an extracellular stimulus results in an increasing frequency, but not amplitude, of intracellular Ca^2+ ^spiking [58,66]. Conversely, the stochastic nature of VEGF-induced Ca^2+ ^oscillations in ECFCs indicates that they cannot be regarded as a simple digital read-out of cell stimulation. More recent studies disclosed that the sub-cellular spatial profile of the Ca^2+ ^transient might be crucial in recruiting specific Ca^2+^-dependent targets by repetitive Ca^2+ ^waves [58]. For instance, Ca^2+ ^microdomains arising within a few nanometers of Orai1, rather than InsP_3_Rs-dependent global Ca^2+ ^oscillations, selectively engage the transcriptional programme responsible for mast cell activation by leukotriene C4 [57,58,70]. Needless to say, the opposite mechanism might well be true in PB-ECFCs, with InsP_3_Rs boosting cell proliferation and tubulogenesis and SOCE being only involved in ER recharging. The next step was to examine the [Ca^2+^]_i _elevation ignited by VEGF in UCB-ECFCs. The higher proliferative response manifested by the latter respect to their peripheral counterparts prompted us to expect a difference in the kinetics of the Ca^2+ ^signal. We were, therefore, surprise to observe a similar pattern of Ca^2+ ^oscillations which were not synchronized between adjacent/neighbouring cells even from the same microscopic field of view (manuscript submitted). A major breakthrough in our studies was the discovery that the Ca^2+ ^response to VEGF does not arise when UCB-ECFCs are stimulated in the absence of extracellular Ca^2+^. In other words, Ca^2+ ^entry plays a primary role in these cells, by triggering their oscillatory activity in the presence of VEGF, while its role in their peripheral counterparts is limited to maintain the Ca^2+ ^transients over time. Similar to PB-ECFCs, however, the Ca^2+ ^machinery involved was placed downstream PLCγ activation, a feature implying InsP_3 _and/or DAG involvement (manuscript submitted). We utilised quantitative real time-polymerase chain reaction (qRT-PCR) analyses and immunoblotting to search for conductances other than SOCE in UCB-ECFCs and found that they express TRPC3 [67]. TRPC3 is as DAG-sensitive, Ca^2+^-permeable channel, which is activated independently on PKC and is implicated in the well known effect exerted by VEGF on cell proliferation, migration and permeability in mature endothelium [54,71]. We exploited the small interfering-RNA technique to genetically suppress its expression and assess its contribution to VEGF-induced Ca^2+ ^oscillations. The results were clear-cut: VEGF does not activate any detectable increase in [Ca^2+^]_i _in TRPC3-deficient cells (manuscript submitted). The signalling transduction pathway recruited by TRPC3-mediated Ca^2+ ^entry was not different from that dissected in PB-ECFCs and involved the interaction between InsP_3_-dependent Ca^2+ ^release and SOCE. Intriguingly, UCB-ECFCs are not endowed with InsP_3_R1, whereas they express the other two isoforms present in ECFCs harvested from PB (manuscript submitted). This feature is rather intriguing, InsP_3_R2 being the most suitable receptor subtype to drive repetitive Ca^2+ ^spikes due to its peculiar InsP_3 _and Ca^2+^-sensitivity [72]. Albeit the triggering role of TRPC3 remains to be fully elucidated, we speculate that TRPC3-induced Ca^2+ ^entry might favour InsP_3 _production by enhancing the rate of PLCγ engagement by VEGFR-2. Accordingly, TRPC3 has been shown to induce elicit translocation towards the plasma membrane and subsequent activation of the Ca^2+^-sensitive PLCγ in antigen-stimulated DT40 B lymphocytes, a process which is suppressed by either pharmacological and genetic TRPC3 down-regulation [73]. We suggest that the amount of InsP_3 _synthesized immediately after VEGFR-2 activation is not sufficient to initiate the intracellular Ca^2+ ^spikes, either because of scarce PLCγ recruitment to the plasma membrane or rapid InsP_3 _metabolism. However, DAG, which is generated along with InsP_3_, gates TRPC3 to provide the source of Ca^2+ ^necessary to further stimulate PLCγ and trigger the first Ca^2+ ^pulse in an InsP_3_-sensitive manner. The following reduction in ER Ca^2+ ^levels will then activate SOCE. This positive feedback between PLCγ, DAG, TRPC3, InsP_3_R and SOCE occurs throughout the oscillatory signal and underpins UCB-proliferation; indeed, the rate of cell growth is dramatically impaired upon suppression of VEGF-induced Ca^2+ ^oscillations (manuscript submitted). Altogether, these findings clearly indicate that juvenile ECFCs may select among a different set of Ca^2+ ^entry/release pathway as compared to their older, i.e. circulating, counterparts. This is the first mechanistic difference ever observed between the two cell types and we are currently investigating how TRPC3 expression impact on their pro-angiogenic activity. Our working hypothesis is that TRPC3-gated Ca^2+ ^inflow contributes to enhance their proliferative response to VEGF, thereby rendering their phenotype more prone to cellular-based strategies than peripheral cells. The rationale behind this speculation is that TRPC3 is selectively coupled to a variety of Ca^2+^-dependent decoders, which include, but are not limited to NF-*k*B, NFAT and extracellular signal-regulated kinase (ERK) [74-76]. For instance, Ca^2+ ^entry through TRPC3 is conveyed to calcineurin to induce the nuclear translocation of NFAT in HL-1 atrial myocytes, whereas Ca^2+ ^inflow via L-type (CaV_1.2_) channels is ineffective [77]. In agreement with these findings, TRPC3 induces the expression of growth-related genes in rat ventricular myocytes, albeit it does not influence CaV_1.2_-paced cell contraction [77]. Therefore, we suggest that TRPC3 is a suitable candidate to reinforce the list of targets that might be exploited to genetically manipulate EPC for CBT purposes.

## Conclusion

Cellular therapeutics is regarded as the most promising strategy for the future of cardiovascular diseases to overcome the limits intrinsic to surgical methods, catheter technologies and pharmacological treatments. Indeed, with the ageing of populations and improvement in survival, increasing numbers of patients are not amenable to revascularisation because of comorbid diseases, unsuitable anatomy or the risk of interventional procedures. The injection of autologous endothelial progenitor cells in clinical practice, however, is still far from coming of age due to the exceedingly number of concerns that have been raised by the lack of therapeutic consistency between pre-clinical studies and clinical trials. One of the issues that must be solved before progressing towards a safe application of stem and progenitor cells in the patients is to understand whether and how is possible to rejuvenate the phenotype of senescent EPCs, which are inadequate to meet the demands of repair and regeneration in diseased vessels. Whereas most of the efforts in the field have been carried out to understand which EPC subset utilize, routes and methods of cell delivery dosing, and timing of intervention, less attention has been paid to the biological mechanisms driving their angiogenic activity. In this regard, the discovery that ECFCs, which represent the most suitable candidates to recapitulate the vascular network *in vivo*, exhibit a different blend of Ca^2+ ^channels depending on their source, i.e. peripheral *vs*. umbilical cord blood, should boost the return to basic research. Indeed, Ca^2+^-permeable channels are master regulator of angiogenesis, but Ca^2+^-mediated responses are tightly associated to specific conductances. In other words, a global elevation in [Ca^2+^]_i _is not sufficient to trigger a given Ca^2+^-dependent reaction, such as gene transcription or an increase in mitochondrial metabolism, if it does not involve the Ca^2+ ^channel intimately associated to this process. Our finding that TRPC3 selectively initiates the pro-angiogenic Ca^2+ ^signal in ECFCs isolated from umbilical cord blood, but not peripheral blood, might contribute to understand their higher sensitivity to VEGF. A large number of approaches are available to transfer angiogenic genes into endothelial committed progenitors [22,36,78]. Future work should be devoted to assess whether the induction of TRPC3 expression into ECFCs harvested from peripheral blood of older patients will be able to rejuvenate their phenotype for therapeutic purposes. This would pave the way for the design of an alternative strategy to rescue ischemic organs without the side effects associated to surgical interventions on ageing subjects.

## Competing interests

The authors declare that they have no competing interests.

## Authors' contributions

FM: conceived the study, analyzed and interpreted the data, drafted the manuscript. SD: conceived the study, critically revised the manuscript. MPC: critically revised the manuscript. SM: critically revised the manuscript. BA: critically revised the manuscript. GG: conceived the study, analyzed the data and critically revised the manuscript. VR: conceived the study and critically revised the manuscript. FT: conceived the study, analyzed and interpreted the data, critically revised the manuscript. All authors read and approved the final manuscript.

## Authors' information

FM: Assistant Professor of Physiology at University of Pavia

SD: Ph.D Student at University of Pavia

MPC: Assistant Professor of Anatomy at University of Naples "Federico II"

SM: Full Professor of Anatomy at University of Naples "Federico II"

BA: Associate Professor of Surgery at University of Naples "Federico II"

GG: Assistant Professor of Anatomy at University of Molise

VR: Staff Physician at IRCCS Policlinico San Matteo, Pavia

FT: Associate Professor of Physiology at University of Pavia

## Declarations

Publication charges for this article were covered by research funds of the project Bando Faro 2012 - Finanziamenti per l'Avvio di Ricerche Originali, cofounded by the Compagnia di San Paolo and by the Polo per le Scienze e le Tecnologie per la Vita of the University Federico II in Naples.

This article has been published as part of *BMC Surgery *Volume 13 Supplement 2, 2013: Proceedings from the 26th National Congress of the Italian Society of Geriatric Surgery. The full contents of the supplement are available online at http://www.biomedcentral.com/bmcsurg/supplements/13/S2
